# Editorial: Editors showcase 2023: insights in stem cell research

**DOI:** 10.3389/fcell.2024.1432127

**Published:** 2024-05-31

**Authors:** Bright Starling Emerald, Francesco De Francesco, Toshiyuki Yamane, Carmen Castro, Daniele Bottai

**Affiliations:** ^1^ Department of Anatomy, United Arab Emirates University, Al-Ain, United Arab Emirates; ^2^ Department of Reconstructive Surgery and Hand Surgery, Azienda Ospedaliera Universitaria Delle Marche, Ancona, Italy; ^3^ Department of Stem Cell and Developmental Biology, Mie University, Tsu, Japan; ^4^ Institute for Biomedical Research and Innovation of Cádiz, University of Cádiz, Cádiz, Spain; ^5^ Department of Pharmaceutical Sciences, University of Milan, Milan, Italy

**Keywords:** stem cell research, embryonic stem cells (ES cells), porcine pluripotent stem cells, human gingival MSCs, MicroRNAs, cancer stem cells, Huntington’s disease

Stem cell research stands at the forefront of biomedical innovation, offering unprecedented opportunities to revolutionise medicine and our understanding of human biology. Stem cells possess unique properties, such as self-renewal and the ability to differentiate into various cell types, making them invaluable tools for regenerative therapies, disease modeling, and drug discovery. This Research Topic will explore key insights driving advancements in stem cell research. Over the past few decades, significant progress has been made in harnessing the potential of stem cells for therapeutic purposes. Pluripotent stem cells, including embryonic stem cells (ESCs) and induced pluripotent stem cells (iPSCs), have emerged as cornerstones of regenerative medicine. ESCs, derived from early-stage embryos, have the capacity to differentiate into any cell type in the body, holding promise for treating a wide range of diseases and injuries. Meanwhile, iPSCs, generated by reprogramming adult cells, offer a revolutionary approach that sidesteps ethical concerns associated with ESCs while providing patient-specific cell lines for personalised therapies. In the review by Neira et al. (Front. Cell Dev. Biol. 2024, 12: 1371240. doi: 10.3389/fcell.2024.1371240), the authors analyse the potential of porcine pluripotent stem cells (pPSCs), including embryonic stem cells (pESCs) derived from pre- and peri-implantation embryos and induced pluripotent stem cells (piPSCs) using a variety of cellular reprogramming strategies. Similarly, Babini et al. (Front. Cell Dev. Biol. 2024, 12:1298007. doi: 10.3389/fcell.2024.1298007) have used, in the cardiology field, human induced pluripotent stem cells (hiPSCs) and the subsequent generation of hiPSC-derived cardiomyocytes (hiPSC-CMs) to recognise the underlying causes of various cardiovascular diseases and identify treatment opportunities which were not possible using *in vitro* or *in vivo* methods.

The therapeutic potential of stem cells spans various medical fields, including neurology, cardiology, oncology, and beyond. Clinical trials exploring stem cell-based treatments, as well as their derivatives, have yielded encouraging results. Della Rocca et al. (Front. Cell Dev. Biol. 2024,11:1260019. doi: 10.3389/fcell.2023.1260019) used the extracellular vesicles produced by human gingival MSCs in an *in vitro* model of cardiomyocytes conditioned by hypoxia, analysing the potential protective and regenerative role through the analysis of the expression of inflammatory, oxidative stress, angiogenesis, cell survival and apoptotic markers, highlighting how these derivatives exerted protection on cardiomyocytes exposed to hypoxic conditions. Koka et al. (Front. Cell Dev. Biol. 2024, 12:1382789. doi: 10.3389/fcell.2024.1382789) analyse the involvement of microRNAs and above all their use as potential interventional treatments for the cytopenias that occur with HIV/AIDS, identifying the miRNA-15a and miRNA-24 secreted by virus-infected CD4^+^ thymocytes and that their differential expression following HIV infection causes indirect inhibition of hematopoiesis. In another manuscript Koka and Ramdass (Front. Cell Dev. Biol. 2023, 11:1296986. doi: 10.3389/fcell.2023.1296986), the same authors analysed progenitor stem cells’ behaviour when exposed to invading pathogens such as HIV-1 and SARS-CoV-2, which cause and extend their damage to other cell subtypes. Knowing the pathogenetic mechanism makes it possible to identify molecular therapies with microRNAs to address cellular dysfunctions and prevent cell loss.

Cancer stem cells are potential therapeutic targets for restraining tumour growth, chemoresistance, and metastasis. Kaur et al. (Front. Cell Dev. Biol. 2024; 12: 1356421. doi: 10.3389/fcell.2024.1356421) investigated the effects of biomaterials targeting the CD47-SIRPα immune checkpoint on breast tumour cells. Using a breast carcinoma stem cell line, the authors compared the effects of a CD47 antibody and a SIRPα-Fc fusion decoy designed to block the interaction between CD47 and SIRPα. The authors demonstrated that the SIRPα decoy exerts an agonistic effect on CD47 signalling and may stimulate proliferation and metastasis pathways in tumour cells. This raises concerns regarding using SIRPα decoys as agents for eradicating tumours.

Huntington’s disease (HD) is caused by the CAG expansion in the huntingtin gene (HTT). Laundos et al. (Front. Cell Dev. Biol. 2023; 11:1252521. doi: 10.3389/fcell.2023.1252521) modulated HTT expression in isogenic human embryonic stem cells to explore its role in HD phenotypes. *In vitro* assays showed HD mutation and HTT depletion produced similar phenotypes. However, reducing wild-type HTT levels did not replicate HD phenotypes, suggesting any loss of function. Mutant HTT in non-HD cells induced HD-like phenotypes similar to HTT depletion, indicating a dominant negative effect. This is supported by the fact that adding wild-type HTT improved HD phenotypes. Together, they suggest a further understanding of this effect could guide clinical strategies to counteract mutant HTT’s impact.

As we delve deeper into the complexities of stem cell biology and its applications, interdisciplinary collaboration and rigorous scientific inquiry will be essential for realising the full potential of this transformative field. By navigating the scientific, ethical, and regulatory landscapes with foresight and integrity, stem cell research promises to unlock new frontiers in medicine and improve countless individuals’ lives worldwide.

